# Insights into the Synthesis of Spiral Beta Zeolite with Enhanced Catalytic Performance in VOC Abatement

**DOI:** 10.3390/molecules29061386

**Published:** 2024-03-20

**Authors:** Chaoqun Bian, Xiaohui Luo, Xiao Chen

**Affiliations:** Pharmaceutical and Material Engineering School, Jinhua Polytechnic, Jinhua 321000, China; jhc0579@163.com (X.L.); morningchenxiao@163.com (X.C.)

**Keywords:** Beta zeolite, spiral morphology, crystallization process, VOCs abatement

## Abstract

The rational synthesis of zeolites with designed morphology is a highly challenging task. In this study, we propose 1,5-bis(methylpiperidine)pentylammonium hydroxide (BMPPAOH) as an organic structure-directing agent (OSDA) based on theoretical calculations. The morphology of zeolite samples is characterized by XRD, SEM, TEM, N_2_ sorption isotherms, and UV Raman spectroscopy. This simple bis-quaternary ammonium salt favored the formation of spiral morphology in Beta zeolite spheres (S-Beta). The crystallization of zeolite in the presence of BMMPAOH is a two-stage process, where nanoparticles agglomerate into spheres in the early stages followed by the emergence of S-Beta crystals with spiral morphology. The synthesized Pt-S-Beta catalysts show higher catalytic activity in VOC abatement compared with other Pt-Beta samples.

## 1. Introduction

Zeolites are crystalline, porous materials with a high surface area, uniform pore channels, high ion-exchange capacities, excellent adsorption capability, and high hydrothermal stability. Due to these unique structural features, zeolites have found many applications in catalysis, gas separation and purification, and sorption [1,2,3,4]. However, the catalytic performance of conventional zeolites is often restricted by their morphology which limits mass transport and utilization of available active sites, especially in the conversion of bulky molecules. One possible solution to this problem is the synthesis of hierarchical zeolites [5,6,7].

Hierarchical zeolites have been the focus of many researchers working on novel catalysts, adsorbents, and other applications [8]. Among them, nanozeolites have emerged as particularly useful materials owing to the high diffusion rate and efficient conversion of sterically hindered substrates [9,10]. Nevertheless, the applicability of nanozeolites is still limited by their complex synthesis and difficulties in their separation from reaction products.

The separation problems could be solved by controlling the crystallization of nanozeolites into the large, ordered hierarchical structures with designed morphology [11,12]. However, these syntheses are still a highly challenging task. Spiral morphology is one of the most favorable zeolite morphologies and has been observed in some compounds and ceramics [13,14]. Tang and co-workers, for the first time, synthesized MTW zeolite crystals (also known as ZSM-12 zeolites, which often exist as intergrown polymorphs of two-members of monoclinic and orthorhombic phases) with a fractal and spiral structure and morphology, but the application of such material has been limited [15].

*BEA-type zeolites, also known as Beta zeolites, are partially disordered structures with a framework made of 3D channels of 12-membered rings with dimensions of 0.66 × 0.67 nm along the [100] plane. This material is well-known for its high catalytic activity in m-xylene isomerization, VOC abatement, etc. [16,17,18,19]. The conventional synthesis of Beta zeolites includes quaternary ammonium ions like tetraethylammonium cation (TEA^+^) as an organic structure-directing agent (OSDA). Other examples of OSDA for Beta zeolite synthesis include polydiallydimethylammonium (PDADMAC) [20], 4,4′-trimethylenebis(*N*-methyl,*N*-benzylpiperidinium) [21], or 3,10-diazoniabicyclo [10.2.2]hexadeca12,14,15-triene-3,3,10,10-tetramethyl-dichloride [22]. Aluminum-rich Beta zeolites can also be obtained under organic template-free conditions [23]. To date, no studies about BEA zeolites with spiral morphology have been reported.

**Figure 1:**
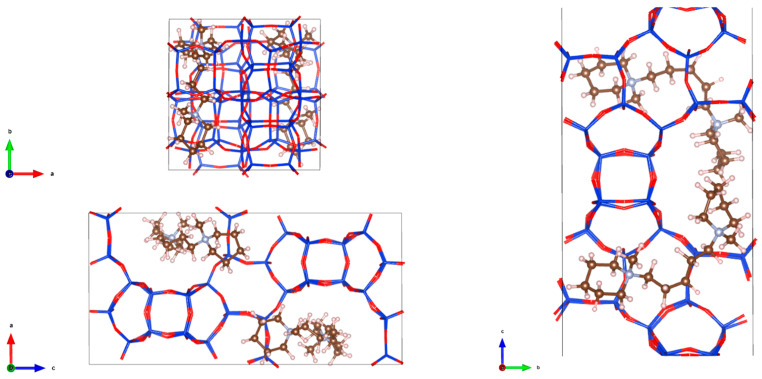
Simulated diagrams of BMPPAOH molecules positions in S-Beta zeolite frameworks.

Herein, we proposed 1,5-bis(methylpiperidine)pentylammonium hydroxide (BMPPAOH) as an OSDA for the synthesis of Beta zeolites with spiral morphology. The preliminary theoretical calculations of interaction energies between BMPPAOH and the zeolite framework (Figure 1) indicated the feasibility of this procedure. We successfully prepared and characterized spiral Beta zeolites (S-Beta) with a high-quality nanocrystalline structure. It was found that the crystallization of S-Beta zeolites is a two-step process where nanoparticles first agglomerate into larger spheres and then form spiral S-Beta crystals. S-Beta zeolites showed higher catalytic performance in VOC abatement compared with other Beta zeolites.

## 2. Results and Discussion

### 2.1. Characterization of S-Beta Samples

The applicability of 1,5-bis(methylpiperidine)pentylammonium hydroxide (BMPPAOH) as the organic structure-directing agent (OSDA) for the synthesis of Beta zeolites is suggested by computational methods. The interaction energies between BMMPAOH and unit cells of Beta zeolites are low, −4.61 kJ/(mol·Si·OSDA), and an average of 1.7 molecules of organic template bind to the unit cell of the Beta zeolite (Figure 1).

**Figure 2:**
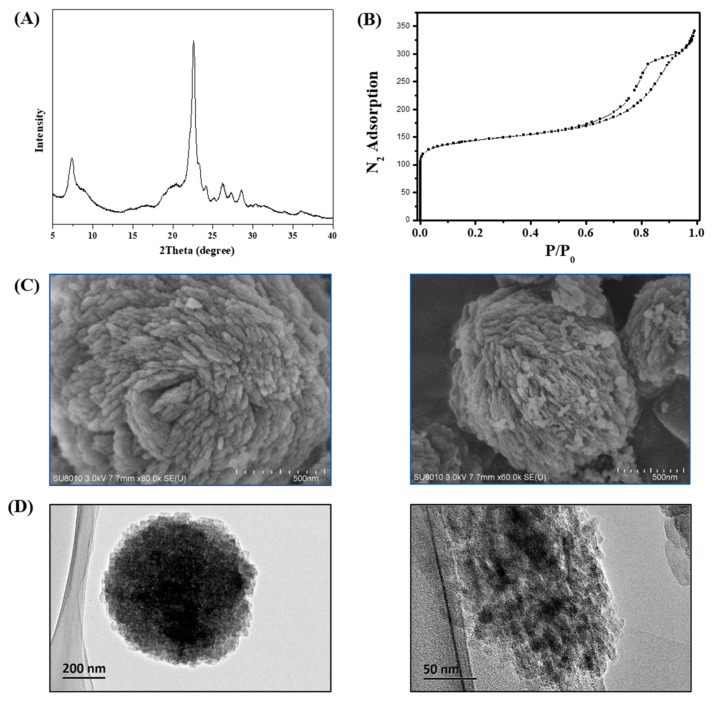
(**A**) XRD patterns, (**B**) N_2_ sorption isotherm, (**C**) SEM images, and (**D**) TEM images of S-Beta samples as synthesized.

The morphology of zeolites highly influences their properties [24,25]. Figure 2 shows the (A) XRD patterns, (B) N_2_ sorption isotherm, (C) SEM images, and (D) TEM images of the S-Beta samples as synthesized. A comparison of the XRD patterns of the S-Beta samples (Figure 2A) and the commercial Beta (C-Beta) zeolite (Figure S1A) clearly shows that the synthesized materials contain structural elements typical of the *BEA structure. The framework type code of as-synthesized samples is *BEA, including mor, bea, and mtw as composite building units, according to the Structure Commission of the International Zeolite Association (IZA-SC) website, [26] and the simulated XRD pattern of this type of zeolite form is shown in Figure S2. The file ID of phase zeolite Beta is PDF#47-0183. The XRD peak of S-Beta around 21.4° decreases and broadens when the morphology changes to spiral. In this experiment, all the experimental factors remain stable when the normal samples (C-Beta) and S-Beta zeolites were under testing. As the morphology changed in this experiment, the possible cause of broad peaks may be ascribed to the smaller particle size of S-Beta zeolites.

The porosity of the S-Beta sample is studied in more detail using the N_2_-adsorption isotherm. Figure 2B shows adsorption curves typical of type-IV plus type-I isotherms. The steep increases in the curve at relative pressures below 0.01 correspond to micropore filling. In addition, a large hysteresis loop suggests the numerous mesopores and macropores in the structure of the S-Beta zeolite and confirms the results of TEM imaging. Accordingly, the adsorption ability of S-Beta is superior compared to that of other Beta samples, as seen from the largest BET surface area (655 m^2^/g) and mesopore area (161 m^2^/g), Table 1 [20,27,28].

SEM images show the special morphology particle size of S-Beta zeolites (Figure 2C and Figure S3). Different from C-Beta (Figure S1C), these zeolites appear as bulky particles containing nanocrystals with a spiral arrangement. The particle size of nanocrystals is between 30 and 60 nm, while bulky particles are around 1 μm in diameter, as shown in Figure S3. The sample morphology is uniform and evenly distributed. This result is in line with the peak broadening observed in XRD experiments.

Nanocrystals were organically bound to bulky zeolite particles, as confirmed by TEM imaging (Figure 2D). After 30 min of S-Beta ultrasonication in ethanol, the bulky particles were still present in the S-Beta, which excluded the possibility of simple mechanical mixing of nanocrystals with the bulky S-Beta particles. Moreover, the TEM images of S-Beta show the presence of a large number of mesopores and macropores, which was ascribed to the aggregation of nanocrystals, in line with the N_2_-adsorption results.

**Figure 3:**
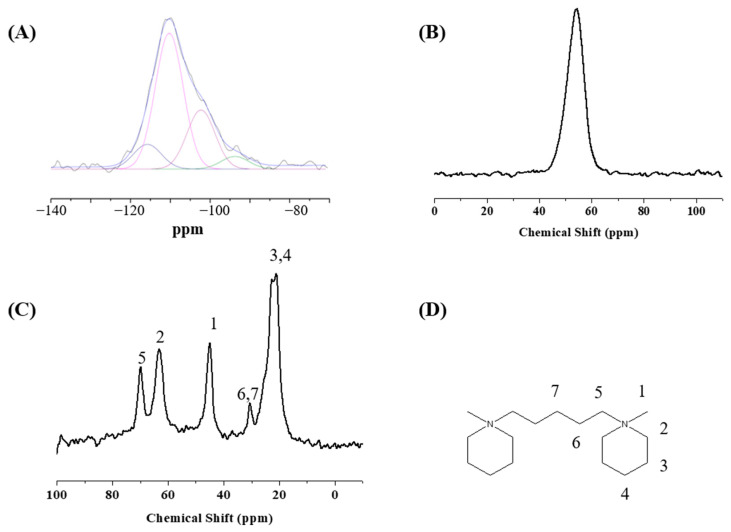
(**A**) ^29^Si, (**B**) ^27^Al, and (**C**) ^13^C solid MAS NMR spectra and (**D**) organic template structure of synthesized S-Beta zeolite.

The structure of S-Beta samples was additionally characterized by ^29^Si, ^27^Al, and ^13^C solid MAS NMR spectra in Figure 3. Three types of Si species, namely, Si(4Si), Si(3Si), and Si(Si), are observed from ^29^Si NMR signals at −110.2 ppm, −104.7 ppm, and −100.6 ppm, respectively (Figure 3A) [29]. A single ^27^Al NMR peak at 56 ppm originates from the isolated Al^3+^ ions within the tetrahedrally coordinated zeolite framework (Figure 3B), while no extra-framework aluminum species have been observed [30]. The stability of the OSDA within the S-Beta zeolite framework is confirmed by ^13^C solid MAS NMR spectra (Figure 3C).

Figure S4 shows the TG-DTA curves of the S-Beta sample. The main weight loss of about 18% is caused by the loss of water and the organic template. The loss under 300 °C is assigned to the water loss, and the loss between 300 and 1000 °C is assigned to the template loss. There are no more peaks above 900 °C, indicating that the silica-alumina zeolite remains stable and that no collapse of framework occurs under the heating process. This result shows great stability of the S-Beta sample.

The crystallization of S-Beta is a complex process driven by the ratio of individual constituents. According to the results in Table 1 and Figure S5, the pure S-Beta zeolite could be obtained only in a narrow area of the K_2_O-Al_2_O_3_-SiO_2_-OSDA phase diagram. At ratios of SiO_2_/Al_2_O_3_ higher than 55, K_2_O/SiO_2_ higher than 0.109, or OSDA/SiO_2_ less than 0.151, the product becomes a mixture of Beta and MTW zeolites. In line with this, an amorphous phase appears when the SiO_2_/Al_2_O_3_ ratio is lower than 25, K_2_O/SiO_2_ is lower than 0.066, or OSDA/SiO_2_ is higher than 0.181. Therefore, the synthesis of zeolites with spiral morphology requires strict control of the amounts of all constituents.

### 2.2. Insights into the Crystallization Process of S-Beta Zeolite

**Figure 4:**
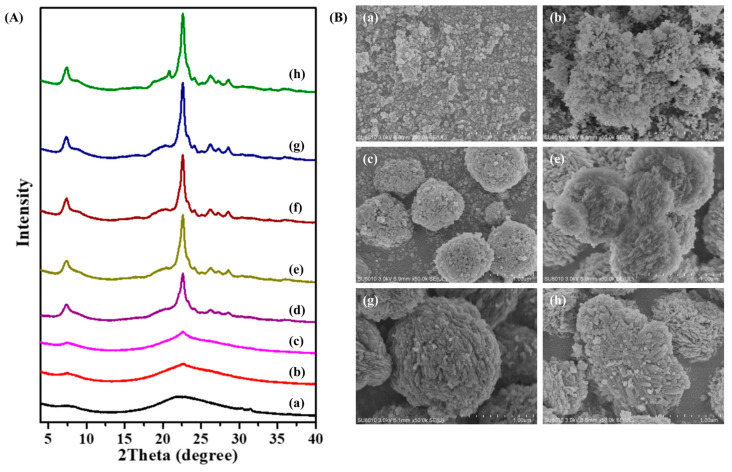
(**A**) XRD patterns and (**B**) SEM images of S-Beta crystallized for (**a**) 2 days, (**b**) 4 days, (**c**) 6 days, (**d**) 7 days, (**e**) 8 days, (**f**) 9 days, (**g**) 10 days, and (**h**) 12 days.

To study the crystallization process and the formation of spiral morphology, the S-Beta zeolites have been characterized at various crystallization times using XRD, SEM, BET isotherms, and UV Raman spectroscopy. The XRD for the first day has no obvious peaks, for the starting materials are amorphous. The SEM images show the irregular particles, confirming the XRD results. The XRD patterns of S-Beta show that the sample stays amorphous during the first 4 days of crystallization (Figure 4(Ab)). Shown in the SEM images, (Figure 4(Bb)), nanoparticles in the mixture agglomerate into larger spheres that later turn into Beta crystals in the early stage of crystallization (2–4 days), which is in line with the XRD results. No obvious Beta crystal came up during this stage. The amorphous nanoparticles agglomerated together rather than crystallizing. After 6 days, the intensity of a series of peaks assigned to Beta zeolites increases. The rate of crystallization is the highest from days 6 to 9. During this process, the nanoparticle of amorphous became crystals of Beta zeolites (Figure 4B). The peak intensity reaches the maximum after 10 days (Figure 4(Ag)), and the perfectly crystalline sample is observed in an SEM image (Figure 4(Bg)). The perfect S-Beta crystals are bulky, spherical particles of about 1μm in diameter decorated with smaller (30–60 nm) S-Beta nanocrystals. Obviously, the crystallization time influences the crystallinity of S-Beta zeolites, and the trend is given in Figure S6. After 12 days of crystallization, the extra peak at 20.8° increases, associated with MTW zeolites, indicating that the S-Beta sample changed its morphology into the MTW type of zeolite. Meantime, the MTW samples consist of bulky zeolite particles with impurities and nanocrystals organized at the surface like a snowflake in the SEM image (Figure 4(Bh)). The results suggest that the formation of spiral morphology is a two-step process: the agglomeration of nanoparticles into larger spheres and the emergence of S-Beta nanocrystals with spiral morphology and a porous structure.

**Figure 5:**
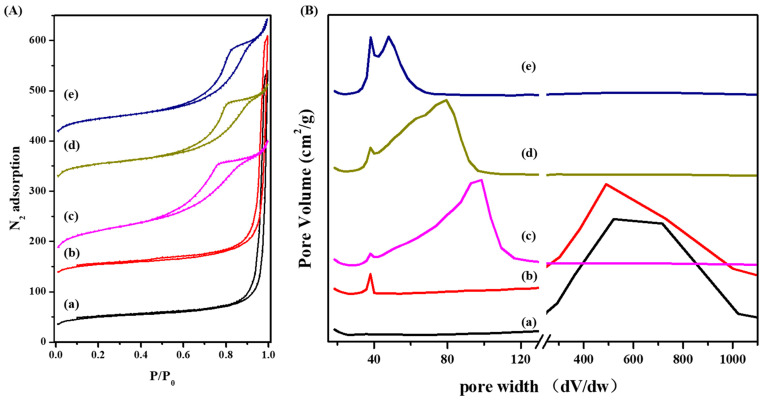
(**A**) N_2_ adsorption isotherms and (**B**) pore width of isotherms of S-Beta crystallized for (**a**) 2 days, (**b**) 4 days, (**c**) 6 days, (**d**) 8 days, and (**e**) 10 days.

The nitrogen sorption data of S-Beta samples were analyzed to provide direct evidence of the porosity and adsorption capacity of zeolite crystals obtained at different crystallization times. The results in Figure 5 and Table 2 show that the adsorption mechanism of the zeolites in the early stages of crystallization could be ascribed to macropores accumulated within the amorphous particles. The microporous volume is 0.04 cm^3^, exhibiting that the mixture is mainly amorphous. This result is in line with the XRD and SEM conclusions. A mesoporous structure in the zeolite spheres becomes significant after 4 days of crystallization, as suggested by the appearance of a low-intensity peak at around 38 nm (Figure 5). In this stage, the amorphous nanoparticles agglomerated together, causing the microporous and mesoporous volumes. Until the sixth day of crystallization the volume of micropores calculated from the *t*-Plot increased from 0.04 cm^3^/g to 0.14 cm^3^/g, which corresponds to the formation of Beta-type zeolites. The maximum microporous volume of 0.18 cm^3^/g is reached after 8 days and could be ascribed to the formation of hierarchical pores with only traces of amorphous regions. After 10 days of crystallization, the perfect crystals are obtained. The crystal growth results in a disappearance of many mesopores and macropores in S-Beta zeolites. This is accompanied by the reduction in total surface area from 693 m^2^/g for S-Beta-8d to 655 m^2^/g for S-Beta. Accordingly, the pore width decreases from 500 nm to 98 nm in the early stages of crystallization due to particle agglomeration and further decreases to 48 nm because of the gradual growth of the Beta crystals in each sphere.

**Figure 6:**
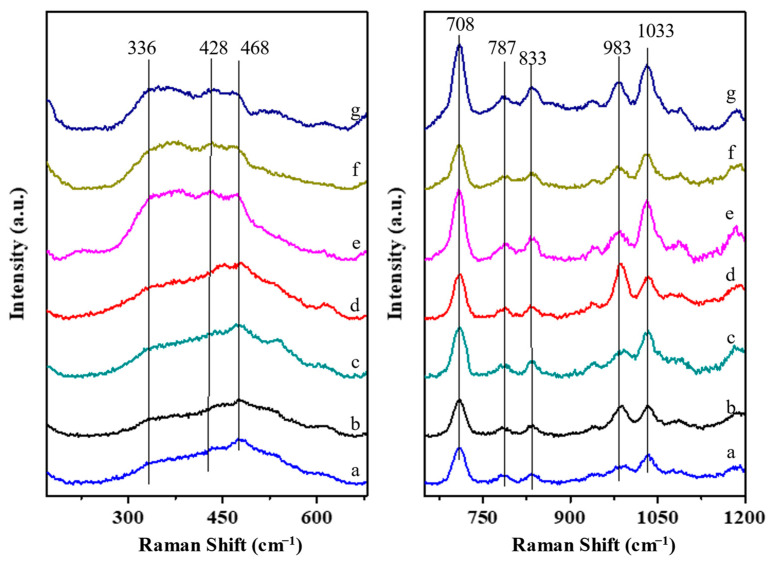
UV Raman spectra of S-Beta crystallized for (**a**) 0 days, (**b**) 2 days, (**c**) 3 days, (**d**) 4 days, (**e**) 6 days, (**f**) 8 days, and (**g**) 10 days.

The UV Raman spectra shown in Figure 6 confirm the changes in zeolite morphology observed at different time intervals by XRD and SEM. There are five main peaks at 708, 787, 833, 983, and 1022 cm^−1^ ranging from 700 to 1200 cm^−1^, associated to T-O species in framework. The bands from 700 to 800 cm^−1^ (708 and 787 cm^−1^) are assigned to Al-O stretches. The bands at 833 cm^−1^ are assigned to Si-O stretches of silicon atoms with two oxygen atoms. The bands at 983 cm^−1^ are assigned to Si-O stretches, and the bands at 1033 cm^−1^ are assigned Si-O stretches of silicon atoms with single oxygen atoms [31,32]. Three broad peaks from 336 to 468 cm^−1^ originate from the S6R (336 cm^−1^) and S4R units (428 and 468 cm^−1^) in the *BEA framework [33,34]. The intensity of these peaks increases at longer crystallization times, which reveals significant growth of the *BEA framework. At first, the peaks change little, reflecting that the main phase is the Armor phase. The intensity mainly increased after 4 days of crystallization, which indicated that the *BEA structure developed. This result is associated with the SEM images and nitrogen sorption data of the S-Beta samples. The broad peaks remain stable after 6 days as the *BEA structure remains in the samples’ framework.

**Figure 7:**
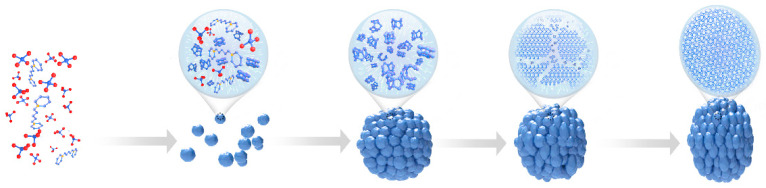
The probable crystallization mechanism of S-Beta.

Based on previously discussed data, we conclude that the crystallization of the S-Beta zeolite is a two-step process: the agglomeration of nanoparticles into larger spheres followed by the emergence of S-Beta nanocrystals with a spiral morphology and a porous structure. The crystallization mechanism is illustrated in Figure 7. At the beginning of the crystallization process, the aggregation of zeolite nanoparticles results in an amorphous rather than crystalline structure. This is ascribed to the high H_2_O/SiO_2_ ratio, leading to nanoparticles as in previous studies [35]. Moreover, the presence of potassium ions facilitates the crystallization of cages and contributes to the formation of nanoparticles [36]. At this stage, bulky spheres form rare helical structural motifs. Afterward, nanocrystals form Beta-type zeolites, and this process is influenced by the organic template, BMPPAOH. BMPPAOH also has a strong directing effect on the formation of twinned crystals with spiral morphology. Similar effects of bis-quaternary, ammonium-type OSDAs have been reported in the literature [15]. Instead of the MTW crystal growth layer by layer, the fractal growth occurs when the nanoparticle turns into Beta crystals in this procedure. The perfectly crystalline *BEA-type zeolite with spiral contours and a hollow structure is formed after 10 days. Finally, after 12 days, fractal MTW zeolite crystals occur in the samples, which is analogous to previously published data on MTW zeolites.

### 2.3. Catalytic Performance of S-Beta in VOC Degradation

**Figure 8:**
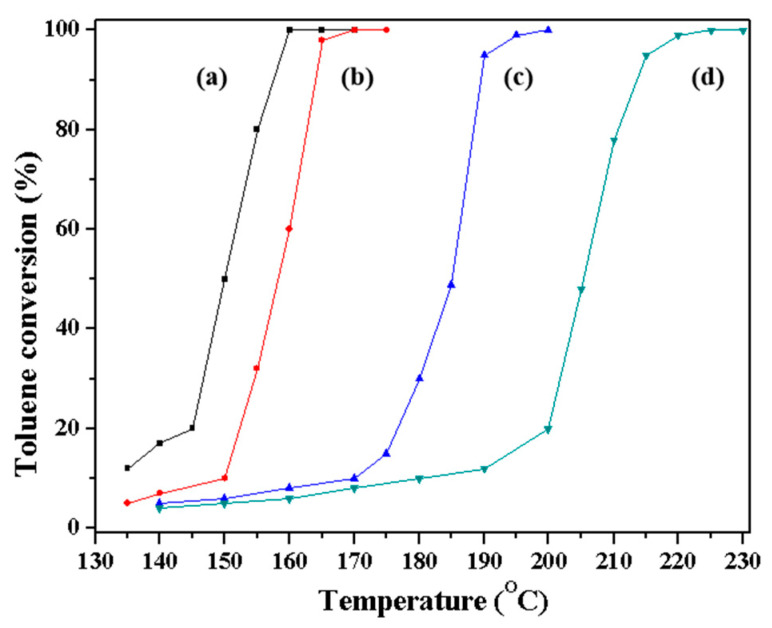
Catalytic combustion of toluene over (**a**) Pt/S-Beta, (**b**) Pt/OTF-Beta, (**c**) Pt/Meso-Beta, (**d**) and Pt/C-Beta catalysts.

According to the references, the type of zeolite, the ratio of Si/Al, the alkali metal species, the amount of Pt ions, and the morphology show significant impacts on the catalytic activity in the VOC abatement reaction. In order to study the effect of morphology, all the samples are K-form Beta zeolite with 1 wt% Pt loading. All the samples have similar Si/Al ratios (around 20). The Beta zeolites with different morphologies are obtained through a different synthesis method according to the references [20,23,28], shown in Figures S1C and S7. The 1% Pt-S-Beta zeolites show catalytic activity in the VOC abatement process, as demonstrated in the toluene combustion reaction (Figure 8). The differences in catalytic activity do not originate from the relative amounts of Si and Al, as all the catalysts have a similar Si/Al ratio. All the Beta samples also get the same amount of K ions as alkali metal species as well as the same Pt loading. Considering the big difference in the morphology, the morphology mainly influences the catalytic results, as reported before. The results presented in Table 3 show that catalytic activity of toluene, expressed as T_5_, T_50_, and T_98_ (the temperature of the conversion at 5% toluene, the temperature of the conversion at 50% toluene, and the temperature of the conversion at 98% toluene) depends on the type of S-Beta catalyst. The temperature of the toluene conversion can represent the catalytic ability in the catalytic combustion of toluene. Lower temperature represents higher catalytic ability. As shown, the catalytic ability of 1% Pt-CBeta > 1% Pt-OTF-Beta > 1% Pt-Meso-Beta > 1% Pt-C-Beta, respectively. The more active catalyst in this process than 1% Pt-Meso-Beta may be ascribed to the combined effects of the mesoporous structure and high surface area [28,37]. The 1% Pt-S-Beta shows better catalytic performance than 1% Pt-OTF-Beta and 1% Pt-Meso-Beta because of the special spiral morphology.

## 3. Experimental Section

### 3.1. Materials

KOH, NH_4_Cl, and Al_2_(SO_4_)_3_·18H_2_O were purchased from Sinopharm Chemical Reagent Co., Ltd. (Shanghai, China). Colloidal SiO_2_ (40% SiO_2_ in water) was supplied by Sigma-Aldrich Co., Ltd. (St. Louis, MO, USA). All chemicals were of analytical grade and were used without additional purification. The deionized water was prepared in the lab. Conventional Beta zeolite (C-Beta, Si/Al = 19), synthesized with TEAOH as OSDA, was ordered from Sinopec (Beijing, China).

### 3.2. Synthesis of 1,5-Bis(methylpiperidine)pentylammonium Hydroxide (BMPPAOH)

A total of 0.5 mol 1,5-dibromopentane and 0.025 mol *N*-methylpiperidine were mixed with 150 mL ethanol in a round-bottom flask. After refluxing and stirring for 12 h at 80 °C, ether was added to the mixture to obtain the white crystals of 1,5-bis(methylpiperidine)pentylammonium bromide. The OH-type anion-exchange resin converted this compound into a 10 wt% solution of BMPPAOH as the final product.

### 3.3. Synthesis of S-Beta, OTF-Beta, and Meso-Beta Samples

In a typical synthesis procedure, 0.164 g KOH, 7.12 g BMPPAOH, and 0.276 g Al_2_(SO_4_)_3_·18H_2_O were added into 3.22 g H_2_O and mixed together. After adding 2.5 g colloidal SiO_2_, the suspension was stirred for 4 h and then transferred into a Teflon-coated autoclave. The temperature in the autoclave was maintained at 160 °C for 10 days, which resulted in the crystallization of S-Beta. The raw product was filtered and washed with deionized water. The same product was calcinated at 550 °C for 5 h, yielding the cal-S-Beta zeolite.

The Meso-Beta zeolites with the mesoporous structure were synthesized using polydiallyldimethylammonium chloride (PDADMAC) as OSDA according to a previously published procedure [20].

The OTF-Beta zeolites were synthesized in the presence of Beta seeds instead of organic template, as reported [23].

All the Beta samples are K-form zeolites with Si/Al ratios around 20.

The Beta zeolite loaded with 1 wt% Pt catalyst was prepared by mixing Beta zeolite and an appropriate amount of H_2_PtCl_6_·6H_2_O aqueous solution by impregnation method. The sample were calcined in dry air at 550 °C for 5 h then reduced to 500 °C for 2 h in a 5% H_2_/Ar stream. Catalysts are labeled as 1% Pt-S-Beta, 1% Pt-C-Beta, and 1% Pt-Meso-Beta.

### 3.4. Characterization

XRD profiles of zeolites were recorded using a Rigaku Ultimate VI powder XRD instrument (40 kV, 40 mA, Tokyo, Japan) equipped with Cu K_α1_ source (λ = 1.5406 Å). N_2_ sorption experiments were conducted at −196 °C (liquid nitrogen) using a Micromeritics APSP 2020M (Norcross, GA, USA) and Tristar system. The *t*-plot and BET methods were used for calculations of the pore volume and surface area of zeolites. The SEM images of zeolites were recorded on a Hitachi SU-1510 microscope (Tokyo, Japan). The composition of samples was analyzed using ICP-OES (Perkin-Elmer 8000, Waltham, MA, USA). The TG-DTA measurements were conducted on a PerkinElmer TGA 7 unit in the temperature range from room temperature to 1000 °C with a heating rate of 10 °C min^−1^. The ^13^C, ^27^Al, and ^29^Si solid magic-angle-spinning nuclear magnetic resonance (MAS NMR) spectra were recorded on a Varian Infinity Plus 400 spectrometer (Palo Alto, CA, USA). The chemical shift of Al(H_2_O)_6_^3+^ was a reference for ^27^Al NMR spectra. TEM experiments were performed on a JEM-3010 electron microscope (110 kV, JEOL, Akishima, Japan), while high-resolution TEM (HR-TEM) images were acquired on a Hitachi HT-7700 microscope. The UV Raman spectra were recorded on a Jobin-Yvon T64000 triple-stage spectrograph (Paris, France) with a spectral resolution of 2 cm^−1^ and UV laser line at 266 nm. The XPS data were obtained using a Thermo Scientific K-Alpha instrument (Waltham, MA, USA) equipped with Al K_α_ X-ray source of radiation.

### 3.5. Computational Method

With Monte Carlo calculation, the quantity of BMPPAOH per Beta unit cell was calculated. Afterward, interaction energies between BMMPAOH and unit cells of Beta zeolites were determined based on Deem’s method [38]. According to this method, energy values were calculated as the average energy from the last 5 ps of a 30 ps molecular dynamics calculation at 25 °C. During the optimization and molecular dynamic simulations, the DREIDING interatomic potential [39] was employed in the molecular dynamics program GULP to calculate these energies [40].

### 3.6. Catalytic Performance Tests

The catalytic performance of Beta catalysts in the degradation of VOCs was analyzed in a continuous-flow fixed-bed micro-reactor at atmospheric pressure, using a quartz tube with an inner diameter of 6 mm. In a typical experiment, 100 mg of 1% Pt-Beta zeolite with particle sizes between 0.40 and 0.60 mm was loaded into the tube and was treated with an airstream at 350 °C for 2 h. The total flow rate of air was 100 mL/min, which corresponds to the space velocity of 60,000 mL/(g·h). The 1000 ppm of toluene gas as a representative VOC was produced by passing the air through a bottle filled with pure toluene and cooled in an ice bath. The gas was diluted to the desired concentration before contact with Beta zeolites using the standard airflow. After the interaction with the catalyst, the amount of toluene in the tail gas was quantified by GC (Fuli, Wenling, China, GC9790) equipped with 19091N-113 INNOWAX capillary column (Agilent, Santa Clara, CA, USA, 30 m × 0.32 mm × 0.25 μm) and FID detector.

The conversion of toluene was calculated from the differences in its concentrations in the inlet and outlet gas. The catalytic activity of zeolites was expressed as T_5_, T_50_, and T_98_ values, which represent temperatures required for the conversion of 5%, 50%, and 98% of toluene. In all experiments, the carbon balance was in the range of 100 ± 5%.

## 4. Conclusions

The rational synthesis of zeolites with designed morphology is a highly challenging task. Herein, we proposed BMMPAOH as a bis-quaternary ammonium-type OSDA for the crystallization of zeolites based on theoretical calculations. This agent improved the crystallinity of zeolites and induced the formation of spiral morphology. Besides OSDA, the crystallization time and the ratio of individual constituents influenced the morphology of S-Beta samples. This simple bis-quaternary ammonium salt favored the formation of spiral morphology in Beta zeolite spheres (S-Beta). After investigation, we concluded that the crystallization of zeolite in the presence of BMMPAOH is a two-stage process, where nanoparticles agglomerate into spheres in the early stages followed by the emergence of S-Beta crystals with spiral morphology. Spiral S-Beta catalysts exhibit enhanced catalytic activity in VOC abatements and could overcome some of the inherent problems of conventional zeolites. This method is a good starting point for the design and synthesis of other molecular sieves with the desired morphology.

## 5. Patents

Patent ZL 2020 1 0272979.3 resulted from the work reported in this manuscript.

## Figures and Tables

**Table 1:** Textural properties of different Beta samples.

Sample	Surface Area (m^2^/g)			V_Micro_ (cm^3^/g)
	Total	Micro	Meso	
S-Beta	655	387	161	0.14
C-Beta [27]	554	328	95	0.13
nano-Beta [28]	833	352	218	0.14
Meso-Beta [20]	483	/	/	0.14

**Table 2:** The changes in textural parameters of S-Beta zeolite samples with crystallization time.

Sample	Surface Area (m^2^/g)			V_Micro_ (cm^3^/g)	Pore Width (nm)	
	Total	Micro	Meso		Meso	Macro
S-Beta-2d	232	83	89	0.04	/	520.6
S-Beta-4d	251	92	95	0.04	37.9	489.9
S-Beta-6d	734	335	218	0.14	38.1	98.6
S-Beta-8d	693	380	145	0.18	38.1	79.4
S-Beta	655	387	161	0.15	38.0	47.9

**Table 3:** The data for the catalytic combustion of toluene over various Beta zeolites.

Catalyst	Activities ^a^		
	T_5_	T_50_	T_98_
1% Pt-S-Beta	130	150	160
1% Pt-Meso-Beta	131	186	195
1% Pt-C-Beta	131	206	220
1% Pt-OTF-Beta	135	157	165

^a^ Estimated from Figure 8.

## Data Availability

Data are contained within the article or Supplementary Materials.

## Supplementary Materials

The following supporting information can be downloaded at: https://www.mdpi.com/article/10.3390/molecules29061386/s1, Table S1: Different conditions on S-Beta crystallization. Figure S1: The (A) XRD pattern, (B) N_2_ adsorption curves, and (C) SEM images of C-Beta zeolite. Figure S2: Simulated XRD pattern of *BEA type zeolite. Figure S3: SEM images of S-Beta zeolite. Figure S4: TG-DTA of synthesized S-Beta samples. Figure S5: XRD of different conditions. Figure S6: The dependence of crystallinity on the crystallization time of the S-Beta zeolite. Figure S7: SEM images of (a) OTF-Beta, (c) Meso-Beta catalysts.
