# Supplementary material for: Insights into the Synthesis of Spiral Beta Zeolite with Enhanced Catalytic Performance in VOC Abatement
Source: Molecules. 2024 Mar 20;29(6):1386. doi: 10.3390/molecules29061386 (PMC10974148; doi:10.3390/molecules29061386)
Supplement: Supplementary file 1 [file molecules-29-01386-s001.zip › molecules-2899705-supplementary.pdf]

Insights into the Synthesis of Spiral Beta Zeolite with Enhanced Catalytic Performance in VOC Abatement

Chaoqun Bian *, Xiaohui Luo and Xiao Chen

Pharmaceutical and Material Engineering School, Jinhua Polytechnic, Jinhua 321000, China; jhc0579@163.com (X.L.); morningchenxiao@163.com (X.C.)

* Correspondence: bian00@zju.edu.cn; Tel.: +86-579-8226-4066

Table S1 Different conditions on S-Beta crystallization.

Run[a]	SiO_2/Al_2O_3	K_2O/SiO_2[b]	OSDA/ SiO_2[b]	H_2O/SiO_2[b]	Products[c]
1	36	0.088	0.161	37	Beta
2	25	0.088	0.161	37	Beta+Amor
3	55	0.088	0.161	37	Beta+ZSM-12
4	36	0.066	0.161	37	Beta+Amor
5	36	0.109	0.161	37	ZSM-12
6	36	0.088	0.181	37	Beta
7	36	0.088	0.151	37	Beta+ZSM-12

[a] Crystallization at 160 °C for 10 days under static state.

[b] The ratio of mixture gel before crystallization.

[c] The phase appearing first is dominant.

Figure S1 The (A) XRD pattern, (B) N_2 adsorption curves and (C) SEM images of C-Beta zeolite.

Figure S2 Simulated XRD pattern of *BEA type zeolite.

Figure S3 SEM images of S-Beta zeolite.

Figure S4 TG-DTA of synthesized S-Beta samples.

Figure S5 XRD of different conditions.

Figure S6 The dependence of crystallinity on the crystallization time of the S-Beta zeolite.

Figure S7 SEM images of (a) OTF-Beta, (c) Meso-Beta catalysts.

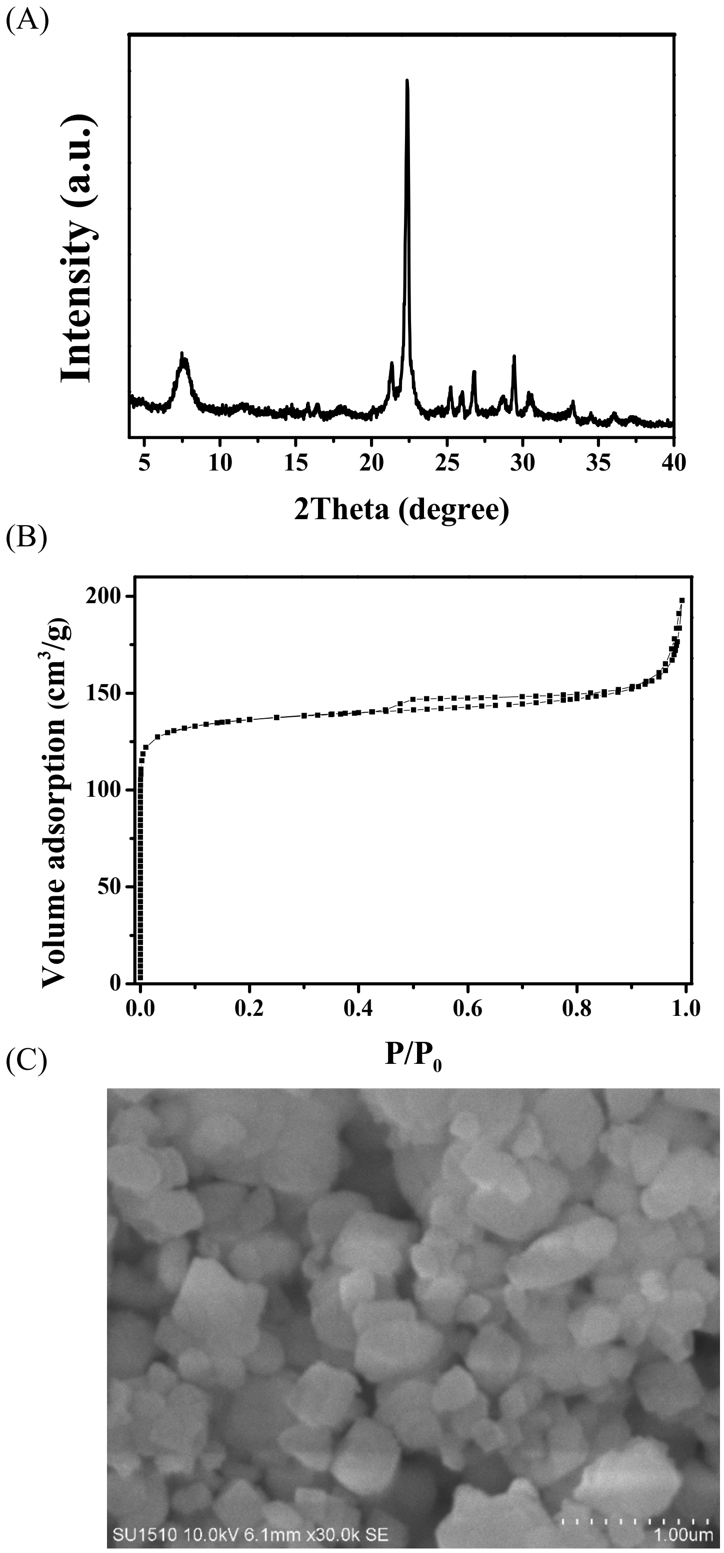

Figure S1 The (A) XRD pattern, (B) N_2 adsorption curves and (C) SEM images of C-Beta zeolite.

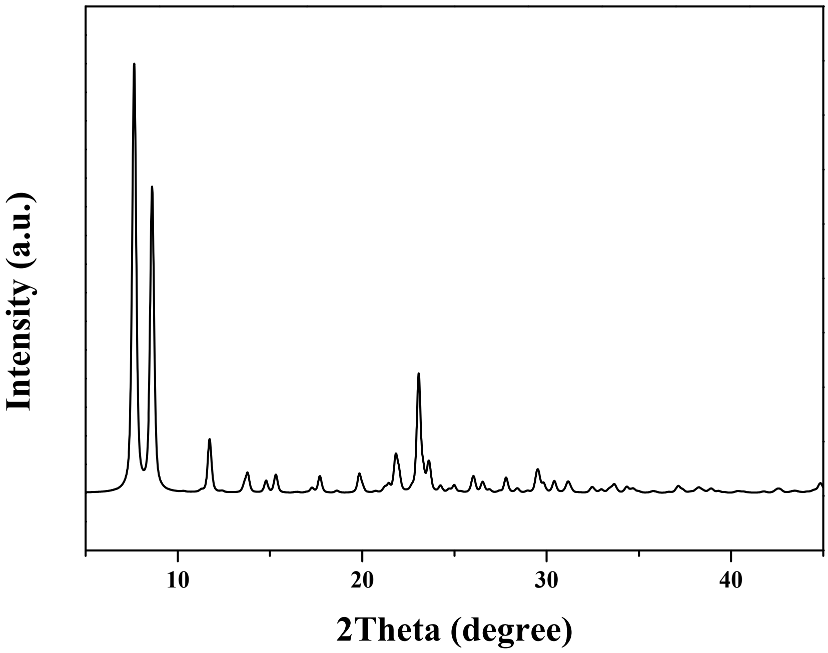

Figure S2. Simulated XRD pattern of *BEA type zeolite.

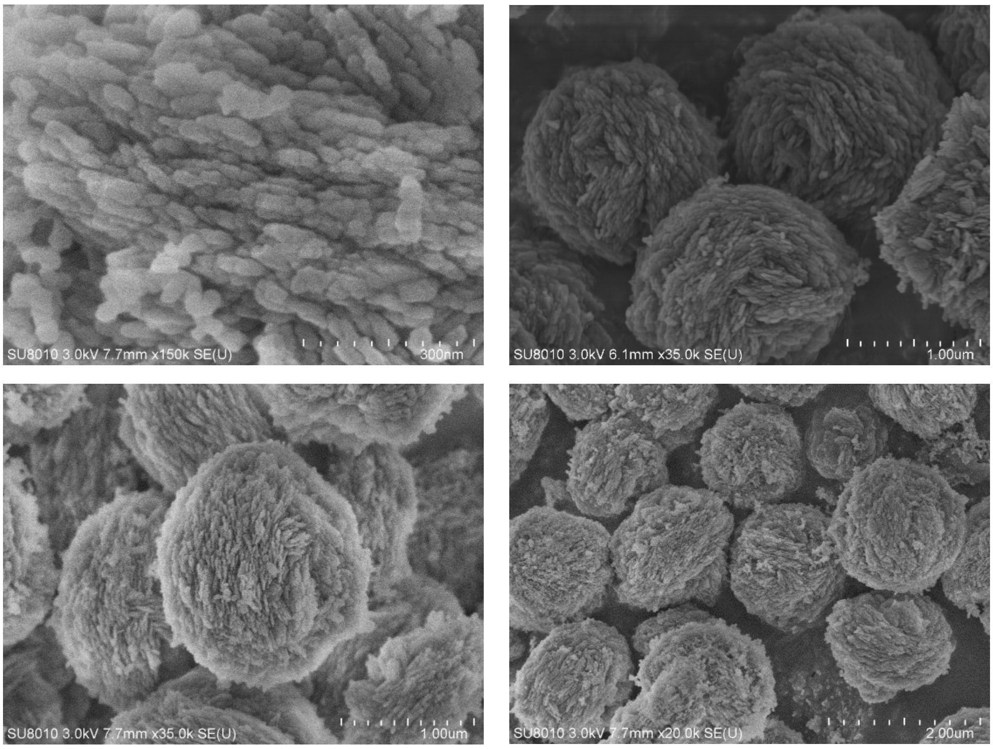

Figure S3 SEM images of S-Beta zeolite.

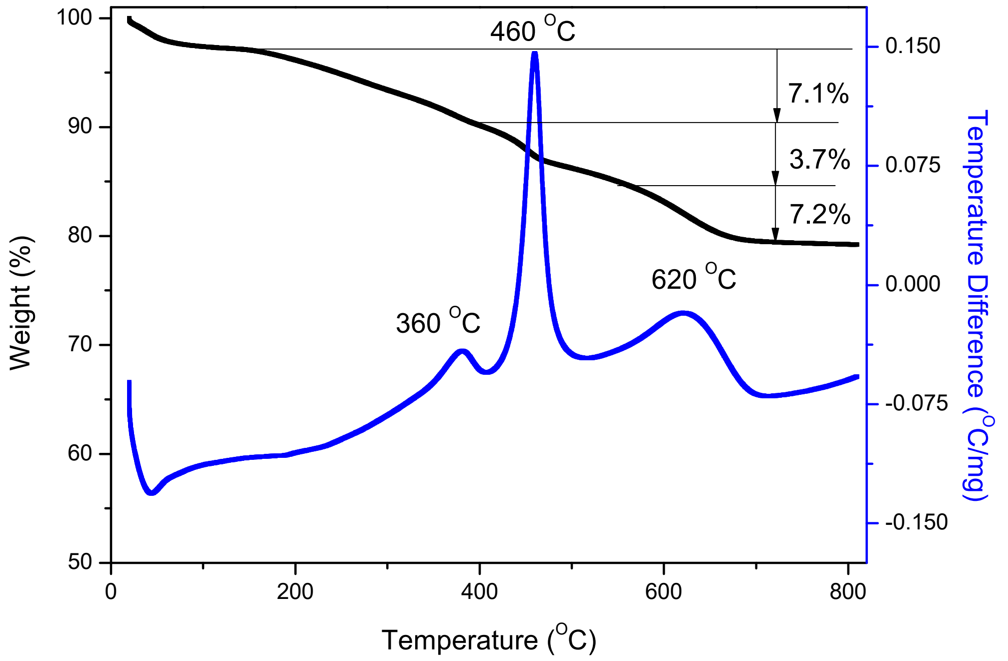

Figure S4 TG-DTA images of S-Beta.

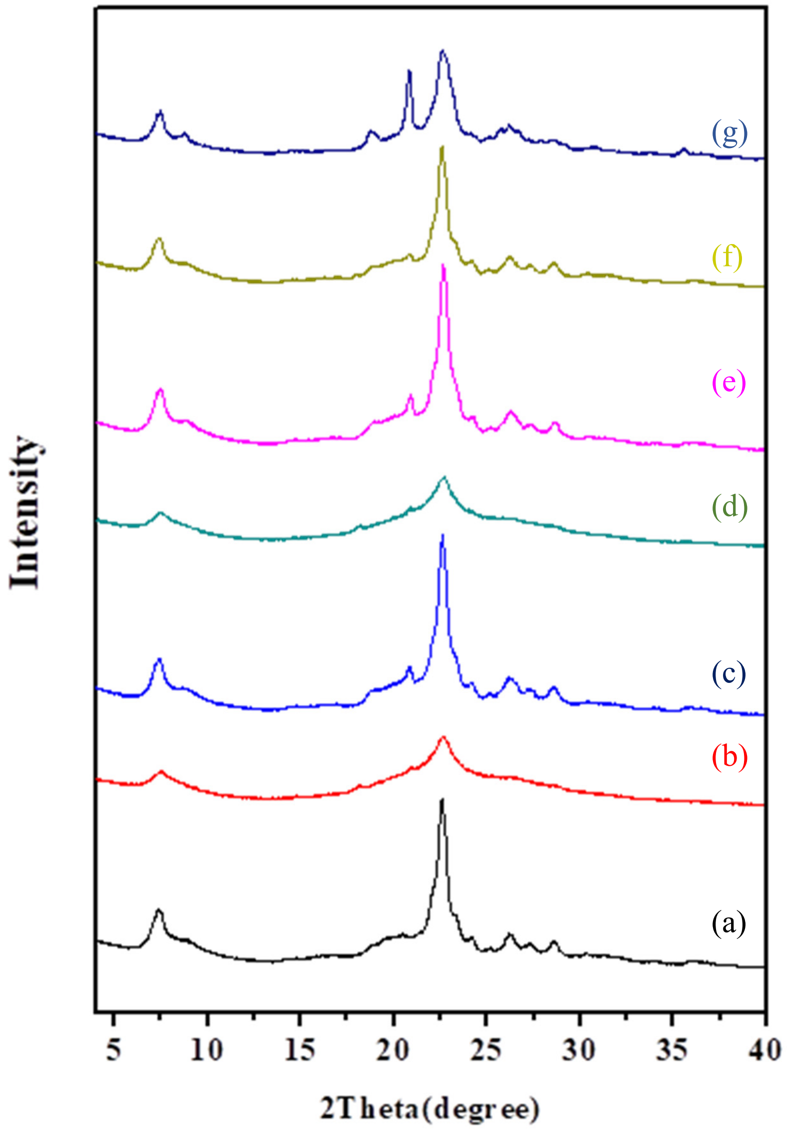

Figure S5 XRD partterns of different conditions in Table S1: (a) Run 1, (b) Run 2, (c) Run 3, (d) Run 4, (e) Run 5, (f) Run 6, (g) Run 7.

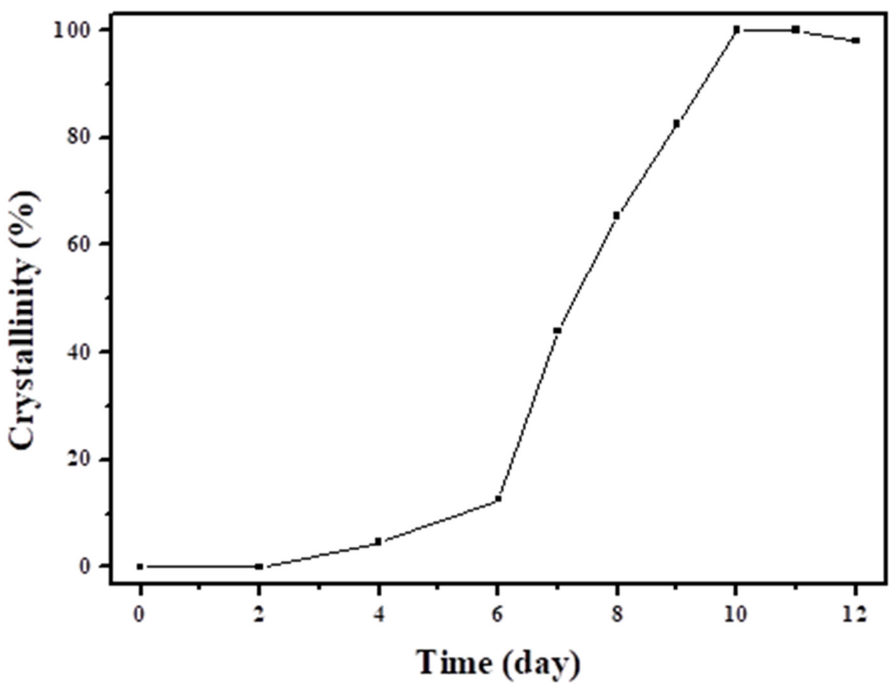

Figure S6 The dependence of crystallinity on the crystallization time of the S-Beta zeolite.

(a) (b)

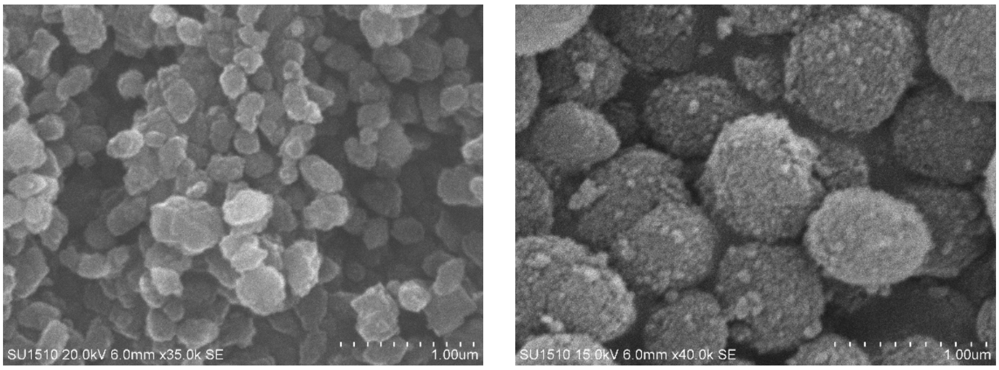

Figure S7 SEM images of (a) OTF-Beta, (b) Meso-Beta catalysts.
